# Association between single nucleotide variants of vascular endothelial growth factor A and the risk of thyroid carcinoma and nodular goiter in a Han Chinese population

**DOI:** 10.18632/oncotarget.15028

**Published:** 2017-02-02

**Authors:** Rui Liu, Lifeng Ning, Xiaoli Liu, Huiping Zhang, Yaqin Yu, Shangchao Zhang, Wenwang Rao, Jieping Shi, Hui Sun, Qiong Yu

**Affiliations:** ^1^ Department of Epidemiology and Biostatistics, School of Public Health, Jilin University, Changchun 130021, China; ^2^ National Research Institute for Family Planning, Beijing 100081, China; ^3^ Jilin Provincial Key Laboratory of Surgical Translational Medicine, China-Japan Union Hospital, Jilin University, Changchun 130033, China; ^4^ Department of Psychiatry, Yale University School of Medicine, New Haven, CT 06511, USA

**Keywords:** papillary thyroid carcinoma, nodular goiter, VEGFA, SNPs, Han Chinese

## Abstract

The aim of the present study was to investigate whether genetic variants in the vascular endothelial growth factor A gene (*VEGFA*) were risk factors for papillary thyroid carcinoma (PTC) or nodular goiter (NG) in Han Chinese. A total of 2,319 subjects (861 PTC patients, 562 NG patients, and 896 healthy controls) were included. Five tag single nucleotide polymorphisms (tagSNPs: rs3024997, rs3025040, rs833070, rs25648, and rs10434) in *VEGFA* were genotyped. SNP rs3025040 T allele was associated with a decreased risk of NG (*P*<0.05). SNP rs3024997 was associated with an increased risk of PTC (*P*<0.05) and NG (*P*<0.001) when an over-dominant model (AA+GG *vs*. AG) was considered. PTC patients carry the less frequent TT genotype (compared to the CC genotype) (*P* <0.05) of SNP rs3025040. Likewise, NG patients have the less frequent TC genotype compared to the CC (*P* <0.05). No significant association of SNPs rs833070, rs25648, and rs10434 with PTC or NG was observed. Haplotypes AT (rs3024997 and rs3025040) and GTA (rs10434, rs3025040, and rs3024997) showed a lower risk for NG (*P* <0.01 and *P* <0.05, respectively), while haplotypes GTT (rs833070, rs3025040, and rs3024997) and GGGT (rs833070, rs10434, rs3024997, and rs3025040) predicted the risk of progression to NG (both *P* <0.05). Haplotype AGAC (rs833070, rs10434, rs3024997, and rs3025040) conferred protection for PTC (*P* <0.05). In summary, this study indicated for the first time that SNPs rs3024997 and rs3025040 in *VEGFA* were significantly associated with PTC and/or NG. Haplotypes of the *VEGFA* may influence the risk of PTC and NG.

## INTRODUCTION

Thyroid cancer is one of the most common endocrine malignancies with a fairly rapid rising incidence throughout the world in recent years [1]. Over the past three decades, the incidence of thyroid cancer has nearly tripled, and almost all of the increase in the incidence of thyroid cancer over the years was due to the increase of the prevalence of PTC [2]. Numerous studies have indicated that complicated interactions between environmental and genetic factors contribute to PTC [3–5]. The risk factors for PTC include old age, extent of lymph node metastases, extra thyroidal invasion, distant metastasis, aggressive histological subtype, large tumor size, and terminal stage [6]. Although the prognosis of PTC appears to be relatively better than other types of cancer, cervical lymph node metastases and aggressive subset are the main contributors to poor prognosis [7]. NG is a common pathology of the thyroid gland, and varies in incidence in different parts of the world [8]. The incidence of thyroid cancer in multinodular goiter is estimated to be 5-10% [9]. The risk factors for NG include goiter size, thyrotrophin, multifocal, heterogeneous, nodular growth pattern, and genetic predisposition [10]. A number of studies have investigated the association between PTC or NG and single nucleotide polymorphisms (SNPs) within or nearby candidate genes [9, 11]. In recent years, many researchers have investigated the genetic susceptibility to PTC, and several susceptibility genes for PTC have been identified [12].

The vascular endothelial growth factor A (VEGFA or VEGF) is one of the most potent inducers of growth factors. VEGF family members include VEGFA, VEGFB, VEGFC, and VEGFD as well as the placental growth factor (PLGF) [13]. The most potent member is VEGFA [14], which acts as a key regulator of both physiological and pathological angiogenesis and modulates proliferation, median survival, and metastasis [15]. The human VEGFA gene is located on chromosome 6, with multiple common SNPs in the promoter as well as the 5′ and 3′ untranslated regions. It is organized into eight exons and seven introns [16]. Evidence of the association between *VEGFA* polymorphisms and susceptibility to cancer (including PTC [17]) has been shown in many previous investigations [18]. However, these sequence variants explain only a small fraction of the estimated heritability of diseases, and the replication of these findings can be difficult.

Due to the conflicting results on the role of *VEGFA* polymorphisms in thyroid diseases [17, 19], more studies are required to clarify these inconsistencies. Additionally, there is no report on the association between thyroid cancer and *VEGFA* polymorphisms in the Chinese population. In this study, we selected five tag SNPs in *VEGFA* to investigate the association between thyroid cancer (PTC and NG) and *VEGFA* polymorphisms in Northern Han Chinese.

## RESULTS

Table 1 showed the age and gender distributions and proportions of the three groups of study participants. In total, 861 PTC patients (209 males and 652 females), 562 NG patients (138 males and 424 females) and 896 control subjects (220 males and 676 females) were enrolled. The median (P25-P75) age of PTC patients, NG patients and control subjects were 44(38-49), 49(42-56) and 43(38-49), respectively. The proportion of females was higher in both case groups, but this difference was not statistically significant (χ^2^=0.023,*P*=0.989). We found statistically significant differences in the median age between cases and controls (*P*<0.001). Multiple comparisons showed that subjects with NG were significantly older than other groups (both *P*<0.001). Genotype distributions of SNPs rs10434, rs25648 and rs3025040 in PTC and NG patients as well as controls fit the Hardy-Weinberg equilibrium (HWE). Genotype distributions of SNP rs3024997 were in HWE only in the PTC group. Genotype distributions of SNP rs833070 revealed deviation from HWE in all three groups. 10% of the samples were randomly selected to check polymorphism genotyping, and no genotyping errors were found.

**Table 1:** Baseline characteristics of subjects

Group	n	Gender (Male/Female)	Age*
PTC	861	209/652	44(38-49)
NG	562	138/424	49(42-56)
Controls	896	220/676	43(38-49)

* Age, median (interquartile range) years.

The genotypic and allelic frequencies of five *VEGFA* polymorphisms are shown in Table 2. The T allele of SNP rs3025040 was less frequent in PTC (17.58%) and NG (16.73%) patients in comparison to control subjects (20.03%) (*P* = 0.050). With multinomial logistic regression analyses adjusted by age and sex, we found a significant association of SNP rs3025040 with NG [OR: 0.81(95%CI:0.67–0.99); *P*=0.042]. No significant differences in allele frequencies of SNPs rs10434, rs25648, rs833070, and rs3024997 (*P*>0.05) were observed between PTC or NG patients and healthy controls. Genotype distributions of SNPs rs3024997 and rs3025040 appeared to be significantly different between cases and controls (both *P* <0.05).

**Table 2:** Genotypic and allelic frequencies

SNP	Genotype/Allele	PTC(%)	NG(%)	control(%)	χ^2^	*P*
rs10434	AA	34(4.01)	22(4.16)	27(3.10)	2.993	0.559
	GA	285(33.65)	171(32.33)	271(31.15)		
	GG	528(62.34)	336(63.52)	572(65.75)		
	A	353(20.84)	215(20.32)	325(18.68)	2.684	0.261
	G	1341(79.16)	843(79.68)	1415(81.32)		
rs25648	CC	656(84.86)	397(87.44)	662(88.15)	-	0.301*
	CT	112(14.49)	53(11.67)	86(11.45)		
	TT	5(0.65)	4(0.88)	3(0.40)		
	C	1424(92.11)	847(93.28)	1410(93.87)	3.733	0.152
	T	122(7.89)	61(6.72)	92(6.13)		
rs3024997	AA	165(19.50)	121(22.45)	150(17.48)	15.997	**0.003**
	AG	418(49.41)	234(43.41)	466(54.31)		
	GG	263(31.09)	184(34.14)	242(28.21)		
	A	748(44.21)	476(44.16)	766(44.64)	0.088	0.957
	G	944(55.79)	602(55.84)	950(55.36)		
rs3025040	CC	574(67.29)	394(70.48)	571(64.09)	10.741	**0.030**
	TC	258(30.25)	143(25.58)	283(31.76)		
	TT	21(2.46)	22(3.94)	37(4.15)		
	C	1406(82.42)	931(83.27)	1425(79.97)	6.010	0.050
	T	300(17.58)	187(16.73)	357(20.03)		
rs833070	AA	204(24.14)	121(22.79)	228(25.97)	3.693	0.449
	AG	146(17.28)	108(20.34)	156(17.77)		
	GG	495(58.58)	302(56.87)	494(56.26)		
	A	554(32.78)	350(32.96)	612(34.85)	1.936	0.380
	G	1136(67.22)	712(67.04)	1144(65.15)		

* Fisher's Exact Test; Bold numbers mean a significant association.

We performed multinomial logistic regression analyses to determine the best inheritance model explaining the association between PTC or NG and the SNPs investigated, and the results are presented in Table 3. Our results revealed no significant association between PTC or NG and *VEGFA* SNPs rs10434, rs25648, and rs833070 (*P*>0.05). In the TT *vs*. CC (PTC cases versus healthy controls) genotypic contrast for the SNP rs3025040, we found a significantly protective effect of the rs3025040 variant on PTC [OR: 0.57 (95%CI: 0.33–0.98); *P*=0.043]. We also found a significantly protective effect of SNP rs3025040 on NG in the TC *vs*. CC genotypic contrast [OR:0.73 (95%CI:0.57–0.93); *P*=0.012]. In the over dominant model (AG *vs*. AA+GG genotypes), we found a significant association between PTC risk and SNP rs3024997 [OR:1.21 (95%CI:1.00–1.47); *P*=0.045] as well as between NG risk and SNP rs3024997 [OR:1.62 (95%CI:1.30–2.02); *P*<0.001].

**Table 3:** Univariable multinomial Logistic Regression analysis of the association between the *VEGFA* polymorphism genotypes and alleles with PTC and NG

SNP	Genotype	Inheritance model	PTC vs. control		NG vs. control	
			OR(95%CI)	P	OR(95% CI)	P
rs10434	GG	Dominant	1.00 (ref)		1.00 (ref)	
	AA +GA		1.15(0.94-1.40)	0.168	1.08(0.86-1.36	0.517
rs25648	CC	Dominant	1.00 (ref)		1.00 (ref)	
	CT+ TT		1.33(0.98-1.78)	0.063	1.08(0.75-1.55)	0.688
rs3024997	AA +GG	Over dominant	1.00 (ref)		1.00 (ref)	
	AG		1.21(1.00-1.47)	**0.045**	1.62(1.30-2.02)	**<0.001**
rs3025040	CC	Codominant	1.00 (ref)		1.00 (ref)	
	TC		0.91(0.74-1.12)	0.378	0.73(0.57-0.93)	**0.012**
	TT		0.57(0.33-0.98)	**0.043**	0.94(0.54-1.64)	0.815
rs833070	AA+ GG	Over dominant	1.00 (ref)		1.00 (ref)	
	AG		0.96(0.75-1.24)	0.772	1.24(0.94-1.64)	0.134

Odds ratio (OR) 95% CI and *P* values was adjusted for age and gender; Bold numbers mean a significant association.

To assess the combined effects of SNPs in *VEGFA* on PTC and NG, we performed haplotype analyses and presented the results in Table 4 and Supplementary Tables 1-3. Our results revealed that a *VEGFA* haplotype, consisting of the minor alleles of SNPs rs3024997 and rs3025040 as well as the major alleles of SNPs rs10434, rs25648, and rs833070, occurred less frequent in PTC and NG cases than in controls (5.91%, 5.26% *vs*. 7.33%, respectively). However, these differences were not statistically significant [PTC vs. Control: OR:0.73 (95%CI: 0.53-1.00), *P*=0.053; NG vs. Control: OR: 0.66(95%CI: 0.44-1.00), *P*=0.050]. Haplotype AT (consisting of alleles of SNPs rs3024997 and rs3025040) showed a protective effect on NG [8.47% (NG) *vs*. 12.00% (Control): *P*=0.009]. Haplotype TG (consisting of allele of SNPs rs25648 and rs833070) played a risk role in PTC [3.97% (PTC) *vs*. 2.64% (controls): *P*=0.049). Haplotype GTT (consisting of alleles of SNPs rs833070, rs3025040 and rs3024997) could predict the risk of progression to NG (*P*=0.025), while haplotype GTA (consisting of alleles of SNPs rs10434, rs3025040 and rs3024997) predicted the protection of progression to NG (*P*=0.011). Haplotypes GGAT and AGAC (consisting alleles of SNPs rs833070, rs10434, rs3024997, and rs3025040) conferred protection to both NG (*P*=0.044) and PTC (*P*=0.027), but haplotype GGGT (rs833070, rs10434, rs3024997, rs3025040) conferred risk for NG (*P*=0.020). Other haplotypes did not show significant associations with PTC or NG.

**Table 4:** Haplotyping analysis of *VEGFA* SNPs in PTC and NG

Block	Haplotype*					Frequency(%)			PTC vs. control		NG vs. control	
	1	2	3	4	5	PTC	NG	control	OR(95%CI)	*P*	OR(95% CI)	*P*
1	G	C	A	C	G	24.33	24.19	21.98	1.00 (ref)		1.00 (ref)	
2	G	C	G	C	G	17.41	16.54	17.82	0.89(0.70-1.12)	0.310	0.89(0.67-1.18)	0.410
3	A	C	G	C	G	11.56	12.66	12.5	0.83(0.64-1.08)	0.175	0.94(0.69-1.28)	0.676
4	G	C	G	C	A	10.28	10.9	9.84	0.94(0.71-1.24)	0.660	1.04(0.75-1.45)	0.816
5	G	C	A	T	G	5.91	5.26	7.33	0.73(0.53-1.00)	0.053	0.66(0.44-1.00)	0.050
6	G	C	A	C	A	5.38	6.14	7.33	0.67(0.48-0.93)	0.016	0.77(0.52-1.14)	0.193
7	G	C	G	T	A	4.44	4.26	4.31	0.93(0.63-1.36)	0.700	1.01(0.63-1.61)	0.976
8	G	C	A	T	A	4.03	3.63	4.74	0.80(0.54-1.17)	0.242	0.67(0.41-1.08)	0.098
9	A	C	A	C	G	4.3	4.76	3.66	1.06(0.71-1.59)	0.758	1.17(0.73-1.88)	0.503
10	A	C	G	C	A	2.49	1.63	2.08	1.12(0.67-1.87)	0.656	0.71(0.36-1.42)	0.333
11	G	T	G	C	A	2.02	1.75	2.51	0.73(0.44-1.23)	0.237	0.65(0.33-1.25)	0.192
12	G	C	G	T	G	1.61	2.01	1.65	0.89(0.49-1.61)	0.698	1.16(0.59-2.28)	0.681
13	G	T	G	C	G	1.61	1.88	1.29	1.12(0.60-2.11)	0.724	1.35(0.65-2.79)	0.420
14	A	T	G	C	A	1.61	1.13	0.65	2.15(0.98-4.72)	0.058	1.79(0.69-4.69)	0.234
15	A	T	G	C	G	1.28	1.13	0.93	1.29(0.63-2.67)	0.484	1.08(0.45-2.61)	0.863
Rare^#^	-	-	-	-	-	1.75	2.13	1.36	1.17(0.63-2.15)	0.623	1.44(0.72-2.88)	0.306

Odds ratio (OR) 95% CI and *P* values was adjusted for age and gender

* SNPs are as follows: 1, rs10434; 2, rs25648; 3, rs3024997; 4, rs3025040; 5, rs833070.

# haplotypes with frequencies<0.01.

## DISCUSSION

By performing the present study, we demonstrated the relationship between five SNPs of the *VEGFA* gene with PTC and NG in a Han Chinese population. In our study, 76% of patients with PTC were female. The median age of 44 years as revealed in this present study is consistent with the findings reported by Shi et al. in 2012 [20]. We found that SNPs rs3024997 and rs3025040 were associated with PTC or NG susceptibility. No association between PTC or NG and other SNPs (rs10434, rs25648, and rs833070) was observed. Haplotype analyses suggested a combined effect of SNPs in *VEGFA* on the risk of PTC or NG.

The basis of some thyroid disorders, including PTC and NG, may reflect the effects of polymorphisms in multiple genes. PTC has relationships with several genes including *FOXE1* [21], *BRAF V600E* [22], and *Fas* [23]. SNPs in *VEGFA* are involved in several types of cancers, such as the bladder [24], esophageal [25], thyroid [11] and gastric [26] cancers. VEGF influences cell angiogenesis, proliferation, and migration, and plays an important role in thyroid cancer cell growth and distant metastasis [27]. The molecular pathogenesis of thyroid cancer involves genetic alterations and their relationship with signaling pathways, including the mitogen activated protein kinase (MAPK) [28]. The MAPK signal pathway is involved in the regulation of cell proliferation and differentiation [29]. Activation of the MAPK pathway mainly drives the development of PTC [30]. The binding VEGFA to its receptor can activate the MAPK pathway [31].

A number of studies have evaluated that age and female gender are risk factors for thyroid cancer [32, 33]. The relationship between *VEGFA* polymorphisms and thyroid diseases had been analyzed in many previous studies. Vural et al. investigated the association between *VEGFA* polymorphisms rs699947, rs833061 and rs2010963 and susceptibility to Graves' disease (GD) risk, and reported that *VEGFA* rs2010963 was a risk factor for GD, and SNP rs699947 C allele was associaetd with elevated autoantibody levels [34]. Marotta et al. examined four SNPs (rs699947, rs833061, rs2010963, rs3025039) in *VEGFA* and demonstrated that the minor homozygous genotypes AA of SNPs rs699947 and CC of SNP rs833061 CC exerted a protective effect on differentiated thyroid cancer (DTC), and haplotype ACG (consisting of alleles of *VEGFA* SNPs rs699947, rs833061, and rs2010963) was protective to DTC [35]. Niccolai et al. reported that SNP rs3025039 (+936C>T) of *VEGFA* was not associated with NG in patients from an area with a mild iodine deficiency [36]. Moreover, Salajegheh et al. revealed that SNPs rs3025039 and rs2010963 (+405C>G) of *VEGFA* might predict the malignancy progresses of thyroid tumor [17]. The study by Hsiao et al [19] suggested that *VEGFA* rs699947 increased the risk of thyroid cancer and lymph node metastasis in males, but not in females. However, these researchers found no significant associations between thyroid cancer risk and *VEGFA* SNPs rs3025039 and rs2010963. Our results showed that individuals carrying SNP rs3025040-T allele had a 0.81-fold decrease risk of developing to NG. The heterozygote AG of SNP rs3024997 was associated with the risk of developing PTC and NG compared with healthy controls. Our study revealed that the TT genotype of SNP rs3025040 protected against the risk of PTC, and the TC genotype of this same variant was protective against NG risk in our study population. There are several explanations for our findings. Sufficient evidence indicates that *VEGFA* plays a complicated and critical role in thyroid disorder [37], and that *VEGFA* polymorphisms can contribute to the interindividual variants in VEGF expression [38]. The inconsistent reports on the role of *VEGFA* in thyroid disorders might be due to the effects of the different polymorphisms in this gene. We had 25.01% to 92.15% power to detect genotype distribution differences between case and control groups. The inadequate power may be the cause of negative results in genetic association studies. Since all patients were recruited in hospital, some potential interfering factors, such as Berkson bias, were not controlled, which might induce bias.

## MATERIALS AND METHODS

### Study participants

We recruited study participants from the China-Japan Union Hospital, Jilin University, Changchun, from August 2012 to December 2014. All cases were unrelated Northern Han Chinese who made clinic visits at the recruitment center. Patients were confirmed by pathologic examinations based on the American Thyroid Association (ATA) guidelines [39]. We excluded specimens confirmed with nodular hyperplasia, anaplastic carcinoma, follicular carcinoma, and follicular variant of PTC. Controls were selected from the general population. They were free from thyroid diseases, cancers, or any other endocrine system diseases. The study included 2,319 participants, comprising 1,423 cases (861 PTC patients and 562 NG patients) and 896 controls.

This study adhered to the tenets of the Declaration of Helsinki. All study subjects made written informed consents before entering the study. Ethics approval was obtained from the Human Research Ethics Committee of School of Public Health, Jilin University.

### SNP selection

*VEGFA* SNPs for the Han Chinese population were chosen from the online HapMap database (HapMap Data Rel 24/phaseII Nov08, on NCBI B36 Assembly, dbSNP b126). Tag SNPs were obtained by implementing the software Haploview version 4.2 (Broad Institute, Cambridge, MA). For all SNPs investigated, we set the threshold for minor allele frequency (MAF) at 0.1, and correlation coefficiency (r^2^) more than 0.8. Five tag SNPs in the *VEGFA* gene were selected, and among them, SNPs rs10434 and rs3025040 were located in the 3’ untranslated region (3’UTR), SNP rs25648 was located in the exon, and the others (rs3024997and rs833070) were located in the introns.

### Genomic DNA extraction

Genomic DNA was extracted from peripheral blood using the DNA extraction kit (Beijing Kangwei Century Biotech Co., Ltd., China). The concentration and purity of DNA samples were determined by spectrophotometer (Shimadzu, Kyoto, Japan).

### SNP genotyping

SNP genotyping was performed using the Sequenom MassARRAY platform (San Diego, CA, USA). Genomic DNA amplification was conducted using specific forward and reverse primer pairs as follows: For rs3024997, the primers were sense 5’-ACGTTGGATGCTCTGTAATGCCACTCTTTG-3’ and antisense 5’-ACGTTGGATGTCAAACACAGTAGGAGGGAC -3’. For rs3025040, the primers were sense 5’-ACGTTGGATGAGATCACAGGTACAGGGATG-3’ and antisense 5’-ACGTTGGATGATCCCCAAAGCACAGCAATG-3’. For rs833070, the primers were sense 5’-ACGTTGGATGTCAGCCTAATGGGATCTCTC-3’ and antisense 5’-ACGTTGGATGAGTTCACAGCACCCGAACAT-3’. For rs25648, the primers were sense 5’-ACGTTGGATGGCACCCAAGACAGCAGAAAG-3’ and antisense 5’-ACGTTGGATGCACAGCCCGAGCCGGAGAG-3’. For rs10434, the primers were sense 5’-ACGTTGGATGTCTCACCTGCTTCTGAGTTG-3’ and antisense 5’-ACGTTGGATGGGCTGCTTCTTCCAACAATG-3’. PCR comprised an initial cycle at 94°C for 15 minutes to perform a hot-start, then 45 cycles at 94°C for 20 seconds, 56°C for 30 seconds, 72°C for 60 seconds, and 1 cycle at 72°C for 3 minutes in the final reaction. Then, the shrimp alkaline phosphatase (SAP) reaction was performed by incubating the PCR product with SAP (Sequenom, Inc., San Diego, CA, USA) at 37°C for 40 minutes, followed by inactivation at 85°C for five minutes. The iPLEX extension reaction was performed at 94°C for 30 seconds and 94°C for five seconds, followed by 40 cycles at 52°C for five seconds and 5 cycles at 80°C for five seconds and at 72°C for three minutes. The PCR products were desalted by the addition of resin in a 384-dimple plate, mixed, resuspended, and centrifuged to separate the extension products from the resin. The completed products were analyzed using the MassARRAY Typer software version 4.0 (Sequenom, USA). Due to the poor quality of some biological samples available for DNA extraction, the genotyping successful rate was not 100% for each SNP.

### Statistical analyses

Conformation with HWE in each group was examined by the Chi-square test. The proportions of genotypes and allelic frequencies were analyzed. Continuous variables (non-normal distribution) were presented as median and percentile values (P25-P75). We analyzed categorical variables using Chi-square or Fisher's exact tests as applicable. Multinomial logistic regression analysis was employed to obtain odds ratios (ORs), 95% confidence intervals (CIs), and *P*-values. Age and sex were adjusted as covariates in the statistical analyses. Haplotypes comprising alleles of the five SNPs were evaluated, and each haplotype frequency was inferred to identify differences between groups. The statistical significance level was set at 0.05, and all statistical tests were two sided. We performed all statistical analyses using R software (version 3.24) (R Foundation for Statistical Computing, Vienna, Austria).

## CONCLUSION

In conclusion, we investigated the association of tag SNPs in *VEGFA* (which is involved in the MAPK pathway) and PTC and NG in a Han Chinese population. We found that *VEGFA* polymorphisms might play an important role in PTC and NG. Carriers of rs3025040-T allele had a decreased risk of developing NG. SNPs rs3024997 and rs3025040 in *VEGFA* were significantly associated with PTC and NG. Combined effect of SNPs in *VEGFA* might influence the risk of PTC and NG. Since the exact mechanism or roles of *VEGFA* SNPs on thyroid disease are still unknown, further studies with larger samples are needed to validate our findings. In addition, sex-specific effects of *VEGFA* SNPs, ethnic differences and gene-environment interaction in the association between *VEGFA* single nucleotide variants and thyroid cancer merit further investigation.

## SUPPLEMENTARY TABLES


