# Supplementary material for: Association between single nucleotide variants of vascular endothelial growth factor A and the risk of thyroid carcinoma and nodular goiter in a Han Chinese population
Source: Oncotarget. 2017 Feb 2;8(9):15838–45. doi: 10.18632/oncotarget.15028 (PMC5362527; doi:10.18632/oncotarget.15028)
Supplement: Supplementary file 1 [file oncotarget-08-15838-s001.pdf]

Association between single nucleotide variants of vascular endothelial growth factor A and the risk of thyroid carcinoma and nodular goiter in a Han Chinese population

SUPPLEMENTARY TABLES

Supplementary Table 1: Association between *VEGFA* haplotypes (2 SNPs) and risk of PTC and NG

Block	Haplotype*		Frequency(%)			PTC vs. control		NG vs. control	
			PTC	NG	control	OR(95%CI)	*P*	OR(95% CI)	*P*
	5	3							
1	G	G	33.67	36.05	34.42	1.00 (ref)	-	1.00 (ref)	-
2	G	A	33.80	32.02	31.75	1.09 (0.92-1.29)	0.316	0.93 (0.77-1.13)	0.473
3	A	G	22.41	21.41	21.82	1.05 (0.87-1.26)	0.624	0.91 (0.74-1.14)	0.419
4	A	A	10.12	10.51	12.01	0.87 (0.68-1.10)	0.231	0.79 (0.60-1.04)	0.087
	2	5							
1	C	G	65.17	64.55	64.88	1.00 (ref)	-	1.00 (ref)	-
2	C	A	26.95	28.41	28.89	0.93(0.79-1.10)	0.404	1.01(0.83-1.22)	0.949
3	T	A	3.91	3.23	3.59	1.08(0.73-1.58)	0.705	0.91(0.56-1.47)	0.701
4	T	G	3.97	3.81	2.64	1.51(1.00-2.29)	**0.049**	1.44(0.89-2.33)	0.140
	3	4							
1	G	C	48.63	47.39	47.89	1.00 (ref)	-	1.00 (ref)	-
2	A	C	33.85	35.47	32.61	1.03(0.88-1.19)	0.740	1.07(0.90-1.27)	0.471
3	A	T	10.25	8.47	12.00	0.85(0.68-1.06)	0.145	0.69(0.52-0.91)	**0.009**
4	G	T	7.27	8.66	7.49	0.96(0.74-1.26)	0.776	1.22(0.91-1.65)	0.179
	3	1							
1	A	G	39.82	38.75	40.89	1.00 (ref)	-	1.00 (ref)	-
2	G	G	39.28	40.70	40.11	1.00(0.86-1.17)	0.966	1.13(0.94-1.35)	0.190
3	G	A	16.41	15.46	15.35	1.09(0.89-1.33)	0.414	1.07(0.84-1.36)	0.576
4	A	A	4.49	5.09	3.66	1.26(0.89-1.80)	0.197	1.44(0.97-2.15)	0.073

Odds ratio (OR) 95% CI and ***P*** values was adjusted for age and gender. Bold numbers mean a significant association.
* SNPs are as follows: 1, rs10434; 2, rs25648; 3, rs3024997; 4, rs3025040; 5, rs833070.

Supplementary Table 2: Association between *VEGFA* haplotypes (3 SNPs) and risk of PTC and NG

Block	Haplotype*			Frequency(%)			PTC vs. control		NG vs. control	
				PTC	NG	control	OR(95%CI)	*P*	OR(95% CI)	*P*
	5	2	3							
1	G	C	A	34.72	34.74	33.50	1.00 (ref)	-	1.00 (ref)	-
2	G	C	G	30.62	31.01	32.09	0.92(0.77-1.10)	0.378	0.96(0.78-1.19)	0.729
3	A	C	G	17.00	16.71	15.94	1.03(0.83-1.28)	0.783	1.05(0.81-1.37)	0.697
4	A	C	A	9.66	10.34	12.15	0.78(0.61-1.00)	0.054	0.81(0.60-1.10)	0.184
5	A	T	G	3.90	3.25	3.65	1.02(0.69-1.52)	0.909	0.87(0.53-1.44)	0.598
6	G	T	G	3.11	3.00	2.18	1.39(0.87-2.23)	0.140	1.35(0.77-2.35)	0.214
Rare#	-	-	-	0.99	0.96	0.49	1.98(0.80-4.91)	0.167	1.94(0.68-5.50)	0.296
	5	4	3							
1	G	C	T	31.77	33.17	32.62	1.00 (ref)	-	1.00 (ref)	-
2	G	C	C	27.95	27.07	24.91	1.15(0.96-1.38)	0.121	1.03(0.84-1.27)	0.753
3	A	C	T	17.19	15.85	16.01	1.10(0.89-1.35)	0.379	0.95(0.74-1.21)	0.655
4	A	C	C	5.71	6.79	7.11	0.83(0.62-1.11)	0.211	0.90(0.64-1.25)	0.531
5	G	T	C	5.65	4.82	6.75	0.86(0.64-1.16)	0.326	0.69(0.48-1.00)	0.050
6	A	T	T	5.35	5.61	5.91	0.93(0.68-1.27)	0.667	0.95(0.66-1.36)	0.761
7	A	T	C	4.50	3.74	4.96	0.94(0.67-1.31)	0.708	0.70(0.46-1.05)	0.085
8	G	T	T	1.88	2.95	1.73	1.12(0.67-1.89)	0.669	1.86(1.08-3.19)	**0.025**
	1	4	3							
1	G	C	G	32.09	32.00	32.67	1.00 (ref)	-	1.00 (ref)	-
2	G	C	A	29.49	30.23	28.76	1.05(0.88-1.25)	0.610	1.03(0.84-1.26)	0.793
3	A	C	G	16.41	15.46	15.28	1.09 (0.88-1.34)	0.441	0.99(0.78-1.27)	0.967
4	G	T	A	10.25	8.51	12.09	0.87(0.68-1.10)	0.238	0.68(0.51-0.92)	**0.011**
5	G	T	G	7.24	8.71	7.52	0.98(0.75-1.30)	0.906	1.23(0.90-1.68)	0.194
6	A	C	A	4.52	5.09	3.61	1.28 (0.89-1.84)	0.177	1.36(0.91-2.04)	0.139
7	A	T	A	0.00	0.00	0.06	-	-	-	-

Odds ratio (OR) 95% CI and ***P*** values was adjusted for age and gender. Bold numbers mean a significant association.
* SNPs are as follows: 1, rs10434; 2, rs25648; 3, rs3024997; 4, rs3025040; 5, rs833070.
# haplotypes with frequencies<0.01.

Supplementary Table 3: Association between *VEGFA* haplotypes (4 SNPs) and risk of PTC and NG

Block	Haplotype*				Frequency(%)			PTC vs. control		NG vs. control	
					PTC	NG	control	*OR(95%CI)*	*P*	*OR*(95% CI)	*P*
	5	2	3	4							
1	G	C	G	C	29.07	29.16	30.37	1.00 (ref)	-	1.00 (ref)	-
2	G	C	A	C	28.53	29.28	25.99	1.15(0.95-1.39)	0.165	1.13(0.90-1.42)	0.301
3	A	C	G	C	12.67	12.41	11.79	1.13(0.88-1.44)	0.352	1.08(0.80-1.46)	0.605
4	G	C	A	T	5.93	5.30	7.42	0.83(0.61-1.14)	0.246	0.73(0.49-1.08)	0.114
5	A	C	A	C	5.67	6.87	7.56	0.79(0.57-1.08)	0.134	0.93(0.65-1.35)	0.713
6	A	C	G	T	4.40	4.34	4.24	1.08(0.74-1.57)	0.691	1.15(0.73-1.81)	0.542
7	A	C	A	T	4.07	3.49	4.66	0.94(0.65-1.37)	0.761	0.72(0.45-1.16)	0.175
8	A	T	G	C	3.67	2.77	3.11	1.22(0.80-1.85)	0.361	0.94(0.55-1.61)	0.820
9	G	T	G	C	2.93	2.89	2.19	1.42(0.88-2.30)	0.151	1.33(0.76-2.34)	0.321
10	G	C	G	T	1.60	1.93	1.62	1.04(0.58-1.87)	0.905	1.24(0.63-2.43)	0.528
Rare#	-	-	-	-	1.47	1.57	1.06	1.46(0.75-2.85)	0.271	1.48(0.68-3.22)	0.320
	5	1	3	4							
1	G	G	A	C	23.80	22.68	21.29	1.00 (ref)	-	1.00 (ref)	-
2	G	G	G	C	19.13	19.69	19.69	0.87(0.70-1.08)	0.202	0.99(0.77-1.27)	0.932
3	A	G	G	C	13.10	13.20	13.37	0.87(0.69-1.11)	0.261	0.94(0.71-1.24)	0.653
4	G	A	G	C	12.48	13.4	12.82	0.87(0.68-1.10)	0.244	0.98(0.74-1.30)	0.901
5	G	G	A	T	5.66	4.74	6.75	0.75(0.55-1.03)	0.072	0.67(0.45-0.99)	**0.044**
6	A	G	A	C	5.47	5.98	6.99	0.70(0.51-0.96)	**0.027**	0.79(0.55-1.13)	0.198
7	A	G	G	T	5.29	5.57	5.95	0.8(0.58-1.11)	0.175	0.92(0.63-1.35)	0.682
8	A	G	A	T	4.49	3.81	5.03	0.81(0.57-1.14)	0.222	0.68(0.45-1.05)	0.085
9	G	A	A	C	4.31	4.23	3.37	1.14(0.78-1.68)	0.487	1.15(0.73-1.80)	0.545
10	A	A	G	C	4.12	2.89	2.76	1.32(0.88-1.99)	0.175	0.97(0.58-1.62)	0.911
11	G	G	G	T	1.91	3.09	1.72	0.99(0.58-1.69)	0.980	1.93(1.11-3.38)	**0.020**
Rare#	-	-	-	-	0.25	0.72	0.25	0.90(0.22-3.62)	0.881	2.58(0.73-9.15)	0.142

Odds ratio (OR) 95% CI and ***P*** values was adjusted for age and gender. Bold numbers mean a significant association.
* SNPs are as follows: 1, rs10434; 2, rs25648; 3, rs3024997; 4, rs3025040; 5, rs833070.
# haplotypes with frequencies<0.01.
